# Dislocation of Humerus of Fourteen Weeks

**Published:** 1861-09

**Authors:** 


### MARINE HOSPITAL—DR. ISHAM'S SERVICE.

Reported by A. D. ROUSE, Hospital Interne.

I. *Dislocation of Humerus of Fourteen Weeks—Reduction.*—.... ......, æt 26, was admitted into the Marine Hospital, May 25. He showed an old dislocation of the right shoulder, which had been reduced a few days after the reception of the injury, but by mishap had been re-dislocated, and remained so for fourteen weeks. It does not seem that the original dislocation was the same as was presented by the patient on admission. The luxation was downwards and forwards; the tumor of the head of the humerus was presented under the pectoral muscles below the clavicle. The shoulder was not as much deformed as when the dislocation is in the axilla. The motion of the arm was restricted, and there was no possibility of elevating the hand to the head, or of bringing the arm forward without great pain. There was a hollow beneath the acromion caused by the absence of the head of the humerus, and flattening of the deltoid. From the statement of the patient, it was seen that the original injury was a luxation into the axilla, and that this more rare position of the bone was consecutive to the former. The patient had never exercised the limb since the accident, and it had acquired no motion in its new position.

The patient having been prepared, at his request reduction was attempted by the ordinary methods, and the surgeon, Dr. Isham, succeeded first in breaking up the newly formed adhesions, but was unsuccessful in reducing the limb without using more force than he felt justified in; the attempt was then abandoned. At the patient's solicitation, another attempt was subsequently made by the professor, with better success, upon the plan of Malgaigne. The patient was laid in a supine position upon a mattrass on the floor, and anæsthesia produced; a folded cloth was placed over the acromion, and the two ends carried to the feet, and held by assistants; an extended band was placed as usual, and the luxated arm slowly and steadily elevated by extension, as much as possible, to make it parallel to the axis of the body, when more forcible extension was used upwards and backwards, the professor strongly bearing against the head of the bone with his palms, to assist it to regain the glenoid cavity. It gradually moved towards its proper position, to which the extension drew it, and the folds of the axilla presented the hollow which usually separates them; the assistants, at the same time keeping up the extension, were desired to slowly bring down the arm, and approximate it to the body. The head of the humerus was thus returned to its place, and secured by properly applied bandages, which were worn for some time. The shoulder recovered in a great degree its roundness, and the motion of the arm could be executed with facility. Some flatness is yet shown, owing to the wasting of the deltoid.

This method of Malgaigne of making extension with the arm raised up, is the application of a general principle to dispose of the bones in such a manner, that they may overlap each other, and that extension may restore the limb to its lost length.

